# Clinical Effectiveness of 3D-Milled and 3D-Printed Zirconia Prosthesis—A Systematic Review and Meta-Analysis

**DOI:** 10.3390/biomimetics8050394

**Published:** 2023-08-27

**Authors:** Harisha Dewan

**Affiliations:** Department of Prosthetic Dental Sciences, College of Dentistry, Jazan University, Jazan 45142, Saudi Arabia; harisha.dewan@yahoo.com; Tel.: +96-65-5491-4588

**Keywords:** monolithic zirconia crowns, 3D printing, milling, trueness, clinical precision

## Abstract

Background: Additive manufacturing (three-dimensional (3D) printing) has become a leading manufacturing technique in dentistry due to its various advantages. However, its potential applications for dental ceramics are still being explored. Zirconia, among ceramics, has increasing popularity and applications in dentistry mostly due to its excellent properties. Although subtractive manufacturing (3D milling) is considered the most advanced technology for the fabrication of zirconia restorations, certain disadvantages are associated with it. Methods: A systematic review was piloted to compare the clinical performance of zirconium crowns that were fabricated using three-dimensional (3D) milling and 3D printing. A meta-analysis was performed, and studies published up to November 2022 were identified. The terms searched were “Zirconium crowns”, “3D printing”, “CAD/CAM” (Computer-Aided Design and Computer-Aided Manufacturing), “Milling”, “dental crowns”, and “3D milling”. The characteristics that were compared were the year in which the study was published, study design, age of the patient, country, the number of crowns, the type of crown fabrication, marginal integrity, caries status, and outcomes. Preferred Reporting Items for Systematic Reviews and Meta-Analyses (PRISMA) guidelines were used to structure this systematic review. Out of eleven hundred and fifty titles identified after a primary search, nine articles were included in the quantitative analysis. The research question based on PICO/PECO (Participant, Intervention/exposure, Comparison, and Outcome) was “Do 3D-printed and milled (P) zirconia crowns and FDPs (I) have a better survival rate (O) when conventional prosthesis is also an option (C)”? The data collected were tabulated and compared, and the risk of bias and meta-analysis were later performed. Only nine articles (clinical research) were selected for the study. Since there were no clinical studies on the 3D printing of zirconium crowns, six in vitro studies were considered for the comparison. Zirconium crowns in the milling group had an average minimum follow-up of 6 months. Results: A moderate risk of bias was found, and survival was significant. A high heterogeneity level was noted among the studies. Marginal integrity, periodontal status, and survival rate were high. Linear regression depicted no statistical correlation between the type of cement used and the survival rate. Conclusions: It can be concluded that the milled crowns had a higher performance and satisfactory clinical survival.

## 1. Introduction

Zirconia-based restorations have become more widely employed in dentistry during the past 20 years. In order to achieve good aesthetic outcomes, patients frequently opt for metal-free restorations, favoring materials with qualities similar to those of natural teeth and similar characteristics, such as light scattering [1]. Zirconia is a notable example of a polycrystalline ceramic, which lacks glassy components and has a thick, cohesive structure that makes it exceptionally difficult to break or fracture [2]. Zirconia was referred to as “ceramic steel” by Garvie et al. (1975) [3] because of these properties. Zirconia can be utilized as a monolithic material since it has the highest strength of any dental ceramic material, offering a number of benefits, including the absence of veneering ceramic chipping and several indications for usage in single, partial, and full-mouth rehabilitation. Additionally, it has a high degree of biocompatibility, little opposing wear, polishability, hardness, low thermal conductivity, and chemical stability [4]. It is known to perform well in the anterior as well as the posterior regions for single as well as the multiple teeth replacements. However, the cost has been a topic of discussion among the various clinical set ups [4,5]. CAD/CAM technologies, which were introduced in the last decade in the dental industry, have lowered the human error in model constructions and crown fabrications. These digital systems are always being improved and developed. Traditionally, Computer-Aided Manufacturing has been carried out using subtractive processes (milling), although additive methods have recently been introduced (printing) [5]. Subtractive procedures mill the prosthesis from an industrial quality blank, either a block or disc, employing numerically controlled computer equipment with multiaxis milling systems. The complicated geometry, cutter tool size, and rotating speed that determine the minimum feature size and surface finish quality are all constrained by the degree of freedom of the cutter and material movement. 

Additive methods use 3D model data to lay down consecutive layers of liquid or powder material that are layered vertically and fused to produce a finished prosthesis. These methods also use extra support structures to fix or support the part throughout the layering process [6]. Additive manufacturing techniques come in many different forms, such as material extrusion, powder bed fusion, plaster-based 3D printing, laminated item production, stereolithography, and polyjet 3D printing [7,8,9]. There are several distinct methods of powder bed fusion, including selective laser melting, electron beam melting, selective heat sintering, and selective laser sintering. 

Similar to selective laser sintering, selective heat sintering uses a thermal print head that is mechanically scanned over the powder bed surface. In selective laser sintering, tiny pieces of plastic, metal, ceramic, or glass are fused together into a mass with a specified 3D “shape”. By applying a layer of powder (plaster or resins) and printing with a liquid binder in the cross-section of the part using an inject-like technique, plaster-based 3D printing (binder jetting) builds the cast one layer at a time. A sheet is attached to a substrate using a heat roller for laminated object manufacture (sheet lamination), and a laser is used to trace the prototype’s desired dimensions and cross-hatch the non-part region to make waste removal easier. The solid layers are created via stereolithography/SLA (vat photopolymerization) utilizing an ultraviolet laser beam that moves over a pool of curable liquid polymer [10]. 

Both the milling and printing methods have benefits and drawbacks [11]. The utilization of a variety of dental restorative materials, the low cost, and the fact that systems are not sensitive to minor imperfections in the solid geometry of a “surface tessellation format (STL)” file are all benefits of milling. However, because of the limitations in accuracy and attainability of an organic shape due to the number of axes the mill allows, the cutters can become dull and inefficient if milling harder materials, and it is challenging to customize color in specific areas until after milling. 

The benefits of printing include practically unlimited design flexibility, the capacity to create complicated structures in a single fabrication cycle, ease of usage, and the ability to change the colors and materials used in different parts of the print [9,12,13,14]. Due to the “step effect” of layered production, which affects surface texture and overall dimensional accuracy of the prosthesis and necessitates post-processing and polishing (which can be corrected by reducing layering thickness to the smallest possible resolution but significantly lengthens the building time of structures), printing has drawbacks such as higher costs for the machines and software, limitations on the materials that can be used, and a high sensitivity to inconsistencies in the input data [15]. 

Using less material waste, passive production that reduces surface microcracking, and fine detail production that allows for the production of a wide range of shapes and sizes that are only constrained by the size of the building chamber of the machine and not the size offered by preformed discs are some of the ways that printing methods solve many subtractive problems [12,16,17,18,19].

Because 3D printing of ceramic dental restorations is still in its infancy, there are not many studies looking at the restorative adaptability, precision, and trueness of 3D-printed full-contour monolithic zirconia crowns. There have not been any substantial reviews that analyze the mechanical and physical performance of 3D-printed zirconia and CAD/CAM-milled prostheses. The novelty of this research lies in the fact that these results are crucial because they can be used to choose the optimum materials and methods for creating crowns and FDPs and hence, the present systemic review was carried out. This systemic review collected and compared studies that were conducted to evaluate the performance of zirconium crowns that were manufactured via 3D printing and milling, and later a meta-analysis was performed to evaluate the impacting factors. 

## 2. Materials and Methods

### 2.1. Study Design

This systematic review was registered in the international prospective register of systemic reviews (PROSPERO, ID: CRD42023403540) and was conducted using “The Preferred Reporting Items for Systematic reviews and Meta-Analyses (PRISMA)” criteria. To stratify the studies and provide a basis for evaluating them, the PRISMA flow system was utilized.

### 2.2. Inclusion and Exclusion Criteria

All the published studies that reported the 3D milling and 3D printing of zirconia crowns performed in human subjects, in vitro studies (since there were no clinical studies for 3D-printed zirconium) studies performed using 3D-printed zirconia, studies published in English, cross-sectional studies, longitudinal studies, and randomized and non-randomized control trials were included in this review. Studies performed in animal subjects, gray literature, meta-analysis articles, narrative reviews, letters to the editors, systematic reviews, studies whose full text was unavailable, abstracts only, and commentaries were excluded from the current review. Table 1 shows inclusion and exclusion criteria for the review.

### 2.3. Search Strategy

A manual and electronic search was conducted, and studies published up to 30 November 2022 were identified. Appropriate key terms (MeSH) and phrases were used, and literature searches were carried out on databases such as Cochrane, PubMed (Medline), Google Scholar, and Scopus. Different keywords were used for the search strategies, such as “Zirconium crowns”, “3D printing”, “CAD/CAM”, “Milling”, “dental crowns”, and “3D milling”. The reference sources of the selected articles were also identified. Details of the search strings used for the systematic search are mentioned in Table 2.

### 2.4. Article Screening

Relevant articles were picked for full-text screening once the eligibility criteria were applied. The paper screening process and eligibility assessment were carried out independently by two reviewers (H.D. and H.C.). In the event of a disagreement amongst the reviewers, the decision was decided by an independent third party (H.N.). The reviewers H.C. and H.N. are mentioned under the Acknowledgements section. Titles were initially screened followed by the abstracts. When the abstracts did not fulfill the criteria of selection, the studies were excluded. All phases of the review involved managing the references using the program Endnote, version X8.0.1. First, repeated references were disregarded. Then, based on the qualifying requirements, two reviewers independently assessed the titles and abstracts. Conflicts were resolved by consensus when lists were compared. Based on the aforementioned selection criteria, the identical two reviewers evaluated the entire texts of articles that might be included in the review. Comparing the lists allowed for disagreements to be resolved through dialogue. Along the review process, a statistic was utilized to gauge the degree of agreement amongst the reviewers.

### 2.5. Data Extraction

The selection of studies, the extraction of data, and the assessment of the search results were carried out independently by two investigators (H.D. and H.C.). Further examination was carried out by retrieving full papers. A Microsoft Excel spreadsheet was customized, and the required data were retrieved from the inclusion criteria, namely, the name of the author and the year in which the study was published, study design, the age of the patient, country, the number of crowns, the type of crown fabrication, and outcomes.

### 2.6. Statistical Analysis

The information gathered was entered into an Excel spreadsheet and analyzed (Microsoft, Redmond, WA, USA). The analysis was valued at 95 percent significance levels. The “Newcastle–Ottawa Scale (NOS) for Quality Assessment” was used to evaluate the quality of the studies (Table 3) by (H.D. and H.C.). Forest plots were presented for age and survival rate, glaze/stain and survival rate, location and survival rate, marginal integrity and survival rate, and bleeding on probing and survival rate to assess study heterogeneity. With the help of the Cochran test (Test Q) and the inconsistency test (I^2^ 50%), the heterogeneity among the studies was measured. Values above 75% (in both tests) were regarded as indicating significant heterogeneity (at the 95% confidence interval (CI)). 

## 3. Results

### 3.1. Selection of the Studies

**Figure 1:**
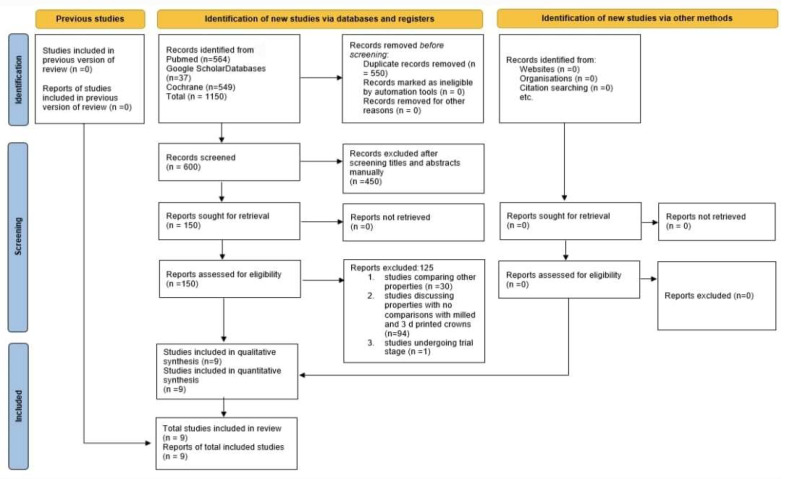
Article selection strategy based on PRISMA guidelines.

Initially, 1150 references were found using the search approach. There were 550 duplicate articles among them, for a total of 600 articles. Following that, 450 items were discarded based on the inclusion and exclusion criteria (reading the title), leaving only 150 articles. A total of 125 items were then excluded, 30 of which were studies comparing other properties, 94 of which were studies discussing properties with no comparisons of milled and 3D-printed crowns, and 1 of which was in the trial stage. A total of nine articles with clinical studies were selected [9,10,11,12,13,14,15,16,17,18,19,20].Only six in vitro studies were found [5,6,7,8,9,11]. Figure 1 depicts the research flowchart and the identification process schematically. A quantitative synthesis of the nine final articles was carried out after a thorough reading of them.

### 3.2. Characteristics of Finalized Studies

The articles that were selected were published between 2004 and November 2022. The total number of clinically placed zirconium units was over 1500, and the most common regions of placement were the posterior regions, which included both the maxillary and the mandibular arches. On average, the patients in these studies were followed up over a year. The articles that were included were one randomized controlled trial [20]; three prospective cohort clinical trials [21,22,23]; one prospective observational case series [24]; two retrospective clinical trials [25,26]; one retrospective observational clinical trial [27]; and one retrospective observational case series [28] (Table 4).

### 3.3. Clinical Studies

The demographics of the patients showed that there was a similar distribution of gender in the clinical studies that were included and that the mean age was over 40 years. Monolithic ceramics were fabricated using various milling methods in all these studies. Resin cements were used for cementation in all the studies except for one, where GIC was used [24]. All the studies reported excellent biological and physical features of the crowns. Bleeding on probing was only reported in four studies and in less than one-third of patients [21,22,23,24,25]. No loss of vitality was reported nor any secondary caries. The most common complaint noted was the wear of the opposite teeth, fractures, and loss of the glaze [21,22,23,24,25,26]. According to Tang et al., polishing the crown again strictly according to the polishing technique, which entailed complete polishing from coarse to fine, decreased the surface roughness [22]. Final extra oral polishing procedures, according to Gunge et al., were carried out using solid polish (Zircon Bite, Dental Ventures of America Inc., Corona, CA, USA). After low-pressure aluminum blasting (0.6 MPa, sandblaster), natural teeth were treated with 10-methacryloyloxydecyl dihydrogen phosphate (MDP) primers [26]. The characteristics are detailed in Table 1.

**Figure 2:**
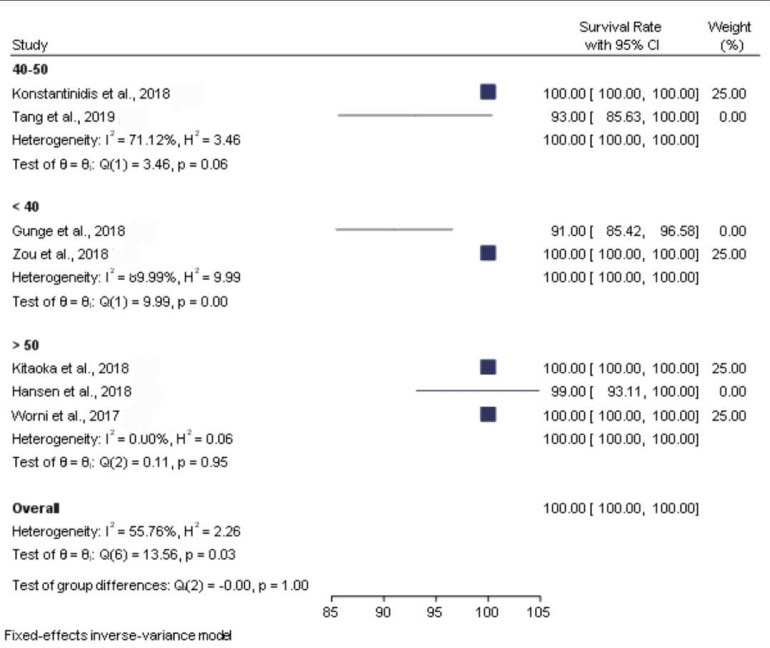
Forest plot graph of the relationship between age (40–50, <40, >50) and survival rate [21,22,23,24,25,26,27], where Q is the Cochran test, I^2^ is the inconsistency test, and H is heterogeneity.

**Figure 3:**
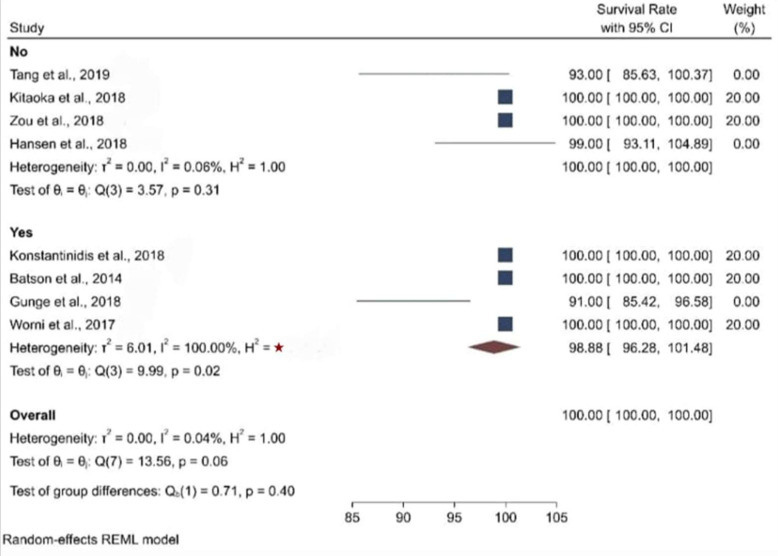
Forest plot graph of the relationship between glaze (Yes or No) and survival rate [20,21,22,23,24,25,26,27,28], where Q is the Cochran test, I^2^ is the inconsistency test, and H is heterogeneity, ★ = 4.01 × 10^6^.

**Figure 4:**
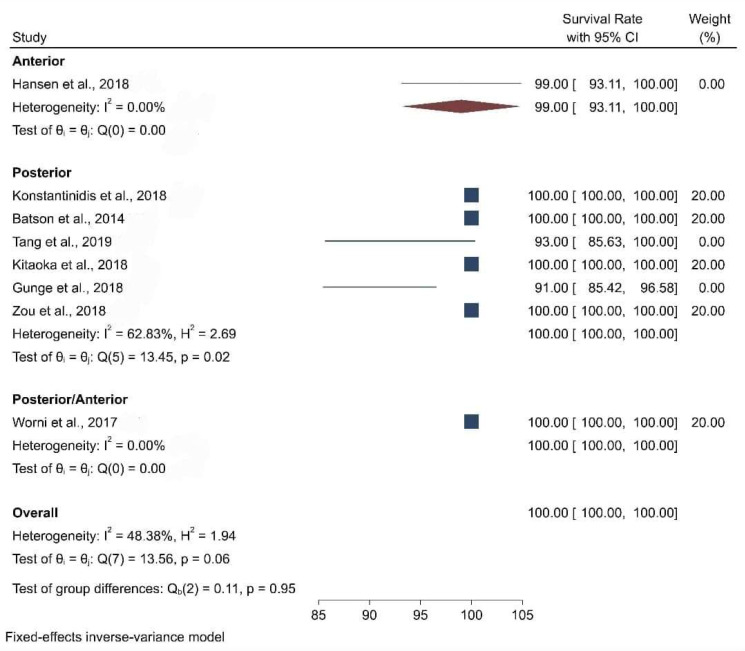
Forest plot graph of the relationship between location (anterior and posterior) and survival rate [20,21,22,23,24,25,26,27], where Q is the Cochran test, I^2^ is the inconsistency test, and H is heterogeneity.

**Figure 5:**
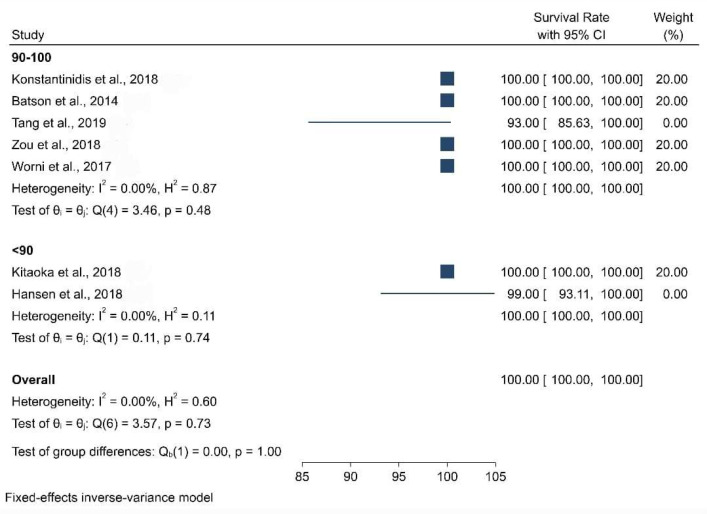
Forest plot graph of the relationship between marginal integrity (%) and survival rate [20,21,22,23,24,25,27], where Q is the Cochran test, I^2^ is the inconsistency test, and H is heterogeneity.

**Figure 6:**
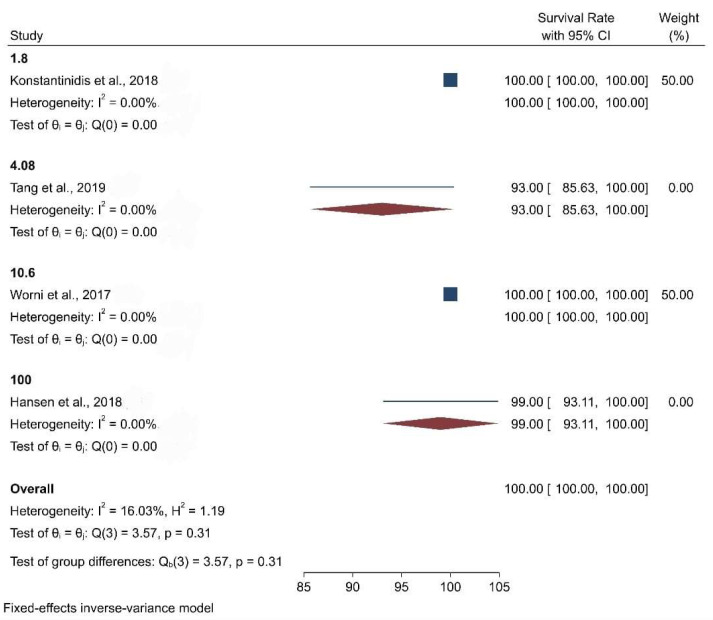
Forest plot graph of the relationship between bleeding on probing (%) and survival rate [21,22,24,25], where Q is the Cochran test, I^2^ is the inconsistency test, and H is heterogeneity.

When the meta-analysis was performed, it was observed that the clinical articles that were included had a moderate level of quality and bias. There was a high level of heterogeneity among the clinical studies when the survival and the biological and physical aspects were considered (Figure 2, Figure 3, Figure 4, Figure 5 and Figure 6).

Figure 2 presents the forest plot for survival rate by age group, which reveals the distribution of survival rate, obtained from studies eligible for the meta-analysis. The pooled survival rate was 100 percent. Furthermore, nominal heterogeneity was found among the studies, with the survival rate ranging from 91 percent (95% CI: 85.42, 96.58) to 100 percent. Subgroup analysis by age group also showed a 100 percent survival rate among individuals with slightly high heterogeneity. 

Figure 3 presents the forest plot for survival rate by glaze/stain, which reveals the distribution of survival rate, obtained from studies eligible for the meta-analysis. The pooled survival rate was 100 percent. Furthermore, nominal heterogeneity was found among the studies, with the survival rate ranging from 91 percent (95% CI: 85.42, 96.58) to 100 percent. Subgroup analysis by glaze/stain also showed a 100 percent survival rate among individuals with slightly high heterogeneity. 

Figure 4 presents the forest plot for survival rate by location, which reveals the distribution of survival rate, obtained from studies eligible for the meta-analysis. The pooled survival rate was 100 percent. Furthermore, nominal heterogeneity was found among the studies, with the survival rate ranging from 91 percent (95% CI: 85.42, 96.58) to 100 percent. Subgroup analysis by location also showed a 100 percent survival rate among individuals with slightly high heterogeneity. 

Figure 5 presents the forest plot for survival rate by marginal integrity, which reveals the distribution of survival rate, obtained from studies eligible for the meta-analysis. The pooled survival rate was 100 percent. Furthermore, nominal heterogeneity was found among the studies, with the survival rate ranging from 93 percent (95% CI: 93.91, 100.00) to 100 percent. Subgroup analysis by marginal integrity also showed a 100 percent survival rate among individuals with slightly high heterogeneity. 

Figure 6 presents the forest plot for survival rate by bleeding on probing, which reveals the distribution of survival rate, obtained from studies eligible for the meta-analysis. The pooled survival rate was 100 percent. Furthermore, nominal heterogeneity was found among the studies, with the survival rate ranging from 93 percent (95% CI: 93.91, 100.00) to 100 percent. Subgroup analysis by bleeding on probing also showed a 100 percent survival rate among individuals with slightly high heterogeneity. 

A decreased survival rate was noted in only two studies [22,26]. The study with the longest follow-up duration and one of the biggest crown samples (n = 148) was that of Gunge et al. [26]. However, Tang et al.’s research, which lasted only 0.8 years, showed a 93% survival rate [22]. 

### 3.4. In Vitro Studies That Included the 3D Printing of Zirconium Crowns

Only six studies that fulfilled the criteria of selection were considered for the present review (Table 5). The study conducted by Wang et al. [7] evaluated the trueness of 3D-printed zirconia crowns. The difference between the 3D-printed and the milled zirconium crowns was not significant, proving that the 3D methods are applicable in clinical setups. Lerner et al. [5] conducted a study in completely different settings using maxillary premolar models, and they found that milled zirconia crowns were as accurate as printed crowns. They also stated that 3D printing can be used for the fabrication of more detailed crowns with all the anatomical structures. Wang et al. [6] evaluated the clinical adaptation and dimensional accuracy of ceramic crowns made using stereolithography methods. They found that zirconium crowns made using this new method of fabrication had similar physical properties. According to Kim et al. [8], there were no appreciable changes between the milled and printed groups in terms of flexural strength (*p* = 0.242) or fracture toughness (*p* = 0.101). The internal and marginal fits of both production procedures were equivalent, according to Abulsaud [9,11], and they were also similar. Compared to the 3D-printed groups, the biaxial flexural strength of the milled group was substantially greater (1507.27 MPa; *p* = 0.01).

## 4. Discussion

The author discovered a dearth of clinical reports of 3D-printed zirconium crowns in this research. There was significant variation in the studies that were included. The sole biological consequence that was triggered in the trials, according to the current systemic evaluation, was bleeding on probing. There was no evidence of the milled crowns being compared to the 3D-printed zirconium crowns. Almost all the clinical studies included in this study mentioned bleeding.

Significant disparities in reported technical problems were observed in three studies [22,24,26], all of which involved a crown fracture. When the study conducted by Gunge et al. [26] was examined, it was discovered that, in addition to being the study with the longest follow-up, it had the largest patient sample. Hence, the survival rate was observed to be low (91.5 percent).

Tang et al. [22] noted that occlusal adjustments might be considered if the patient complains of pain soon after crown placement. It was also noted that wear and pain in the opposite teeth may be experienced after zirconium crowns are placed [29,30,31,32,33,34,35,36,37]. Hansen et al. [24] stated that these crowns should be used with caution in patients with bruxism and other parafunctional habits.

Fractures may be related to excessive tightness or flaws in the crown margins. Low-temperature degradation may also be linked to this sort of failure [37,38]. Nakamura et al. [39] stated that fractures are more evident when there is greater wear. It was concluded from the studies that thorough polishing had lowered the propagation of micro-fractures and, hence, the chance of the crowns fracturing [20,21,22,23,24,25,26].

Different milling systems were employed in the studies, and the type of cement used varied, potentially interfering with the marginal adaptation of the crowns. Despite the fact that the average preservation value of marginal integrity was high (86.09 percent), it dropped in the study conducted by Hansen et al. [24] (31.6 percent). In this study, the nature of the cement and the methods followed when the cementation of the crown was carried out were not evaluated, which may have impacted marginal integrity. In this study’s meta-analysis, a no statistical significance was found between the survival rate with the average years of follow-up and the type of cement used. Similar observations were made in the study conducted by Boitelle et al. [40].

It is now well established from the previous studies that zirconium causes antagonist wear; this is also reinforced by the present systemic review. However, the wear caused by monolithic zirconia is lower than that caused by other ceramics. Hence, these are preferred to other variants.

The literature on the aesthetic aspect of zirconium remains inadequate. The evaluation of the MZ color, according to Worni et al. [25], is complicated by a number of factors. We can reference the location’s illumination conditions, adjacent reconstruction hues, and/or genuine teeth as examples. Haff et al. [41] found that monolithic crowns had a 45 percent acceptable color, which may be due to the variation in the color of the natural adjacent tooth and the new restoration. Polychromatic zirconia discs/blocs are now available on the dental market, and they may help with cosmetic results, although more clinical studies are needed.

There were various limitations and/or consequences of this systematic review. The technique variability, multiple commercial brands, the lower number of patients, and the difference in the follow-up duration were major hindrances in conducting the meta-analysis. In addition to these reasons, there was no comparable method for the calculation of the functional or the aesthetic features in the clinical studies, as well as for the in vitro studies. A moderate risk of bias and quality was found. Visual appeal is regarded as a crucial element in patients’ decision and contentment. Moreover, it promotes the adoption of innovative technology and its triumph in society. Color stability was the only aesthetic component that was analyzed. Only in vitro studies had analyzed functional features. Functional analysis significantly affects the reliability of the findings of the research.

The systematic review’s studies all have a moderate quality of assessment according to the Newcastle–Ottawa Scale, which suggests a moderate likelihood of bias. A limitation of the studies included in this meta-analysis is the small number of patients involved (not representative of the general population) and the crowns studied. However, given that not all studies reference the workflow restriction, it is crucial to include it.

Nonetheless, because not all studies refer to the impression conditions, it is vital to mention the workflow restriction. In general, the impressions employed were traditional, as four of the nine studies analyzed mentioned [21,22,23,26]. The impression circumstances were only mentioned in one article [24]. When compared to traditional modeling techniques, the intraoral scanners had greater efficiency [42,43,44,45].

Zirconia is reportedly the greatest all-ceramic material, despite the fact that the variety of available ceramics has significantly enhanced its properties, according to Miyazaki et al. Therefore, the use of zirconia has potential given the swift development of materials and processing technology. However, additional research and clinical assessments are required [42]. This is also in line with a systematic review [46], which suggests zirconia is a suitable clinical material for molar zones (zones with higher occlusal forces). There are still some issues that need to be evaluated clinically, such as aesthetics (connected to color and its antagonists), long-term chemical stability, and related clinical wear [46].

Belli et al.’s retrospective study [28], which examined the survival rates of 35,000 posterior ceramic restorations, came to the conclusion that the fracture rate at 3.5 years was 1.4%. Additionally, it was demonstrated that within the first 8.5 months of placement, zirconia restorations were performed clinically without experiencing any sort of failure. In a systematic analysis of several ceramic varieties, Carvalho et al. [43] found that ceramics manufactured with a glass matrix had a statistically significant lower failure rate than polycrystalline ceramic restorations (*p* = 0.001; 1.18% versus 3.22%).

Similar findings from a meta-analysis study [46] were obtained, with fixed partial prostheses made of zirconia–ceramic having a survival rate of 95.4% and metal–ceramic crowns having a survival rate of 96.9%, with no discernible differences between the materials.

The survival rate of fixed partial prostheses made of zirconia–ceramic after 5 years, however, is much lower than those made of metal–ceramics (92.1% and 94.7%, respectively), as per the literature [47]. Although most all-ceramic restorations have survival rates that are similar to those of metal–ceramic restorations, alumina and leucite (or lithium disilicate) have survival rates that are greater than those of zirconia and metal–ceramic at 96% and 96.6%, respectively. This is consistent with the results of another comprehensive review [48], which showed that single lithium disilicate crowns had a high survival rate (97.8%) after five years of use. Despite having outstanding aesthetic qualities, feldspathic ceramics had a reduced survival rate (90.7%), which restricts their use to the posterior sector [47].

Another study published in 2014 [49] found that zirconia crowns outperformed metal–ceramic, zirconia with a ceramic covering, and lithium disilicate monolithic crowns in terms of fracture resistance. However, it was found in this same study that crowns with a thickness of 1.0 mm exhibit clinical outcomes comparable to those of metal–ceramic crowns. The observed average survival rate for this study was 98.15%, and the average follow-up time was 1.07 years. This is a positive outcome, despite the short average follow-up time.

This material might be a viable option for the restoration of single crowns, particularly in the posterior sector, but the great number of limitations inherent to this work must be noted, particularly those related to the lack of well-standardized methods and the reduced number of studies and controlled protocols, the reduced sample size, high heterogeneity, the reduced follow-up period, and the overall survival rate of zirconia monolithic restorations made with CAD/CAM technology. Clinical trials generally have a comparatively brief monitoring period and may undervalue possible advantages of the treatments examined, as well as overlook risks, which may take significantly more time to surface. Extended monitoring of trial participants following the conclusion of the planned trial duration can yield crucial data on both effectiveness and safety results. Studies using a smaller sample than required lack enough statistical power to address the main research query, and a statistically insignificant outcome may be solely due to an insufficient sample size. To thoroughly document the potential advantages of monolithic zirconia and establish its superiority when compared to other treatment options, additional studies, ideally long-term randomized controlled trials with an adequate sample size, are necessary. Methodological variation produces diversity/heterogeneity by introducing biases that can influence the results of studies in different ways. The random-effects combined estimate will accurately estimate the average treatment effect only if the biases are evenly distributed, resulting in a combination of both over- and underestimations of the effect. Heterogeneity should be explored further via subgroup analysis or meta-regression.

## 5. Conclusions

It is possible to draw the following conclusions within the scope of this review:

1. Milled and 3D-printed zirconium crowns have shown superior biological characteristics.
2. Zirconia crowns made using additive and subtractive manufacturing methods both had a similar internal fit and marginal adaptation.
3. Zirconia crowns that are 3D-printed or milled can be used as alternatives to traditional prosthetics.
4. Additional in vitro and in vivo investigations are required to assess the mechanical and optical qualities of 3D-printed zirconia crowns, among other factors.
5. Long-term studies with a greater sample size utilizing diverse production procedures are needed to thoroughly establish the potential benefits of zirconia and to assert its superiority over other treatment options.

## Figures and Tables

**Table 1:** Inclusion and extrusion criteria.

Inclusion Criteria	Exclusion criteria
All published studies that reported the 3D milling and 3D printing of zirconia crowns performed in human subjects	Studies performed in animal subjects
In vitro studies (since there were no clinical studies for 3D-printed zirconium) performed using 3D-printed zirconia	Gray literature
Studies published in English	Meta-analysis articles, Narrative reviews, Systematic reviews
Cross-sectional studies	Letters to the editors
Longitudinal studies	Studies whose full text was unavailable, Abstracts only
Randomized and non-randomized control trials	Commentaries

**Table 2:** Search terms and strategy for the electronic databases.

PubMed	((Monolithic Zirconia Crowns) OR (Crowns)) OR (Dental Porcelain) OR (Dental Ceramic) OR (Zirconia) OR (Dental Prosthesis) OR (Dental Crown) OR (Ceramics) AND (CAD CAM) OR (Subtractive Manufacturing) OR (3D milling) OR (Digital one-piece casting) AND (3D printing) OR (Printing three dimensional) OR (3D printing using zirconia) OR (Direct inkjet printing) OR (Stereolithography) OR (Additive manufacturing technologies) OR (Additive technology) AND (Marginal adaptation) OR (Biaxial flexural strength) OR (Colour infiltration) OR (mechanical properties) OR (Strength) OR (Marginal fit) OR (Trueness) OR (Clinical precision)	564
Google scholar	Monolithic Zirconia Crowns, Crowns, Dental Porcelain, Dental Ceramic, Zirconia, Dental Prosthesis, Dental Crown, Ceramics, CAD CAM, Subtractive Manufacturing, 3D milling, Digital one-piece casting, 3D printing, Printing three dimensional, 3D printing using zirconia, Direct inkjet printing, Stereolithography, Additive manufacturing technologies, Additive technology, Marginal adaptation, Biaxial flexural strength, Colour infiltration, mechanical properties, Strength, Marginal fit, Trueness, Clinical precision	37
Cochrane	ID Search Hits #1 MeSH descriptor: [Crowns] explode all trees 864 #2 MeSH descriptor: [Dental Porcelain] explode all trees 380 #3 MeSH descriptor: [Ceramics] explode all trees 684 #4 MeSH descriptor: [Computer-Aided Design] explode all trees 360 #5 MeSH descriptor: [Printing, Three-Dimensional] explode all trees 114 #6 MeSH descriptor: [Stereolithography] explode all trees 2 #7 MeSH descriptor: [Dental Marginal Adaptation] explode all trees 535 #8 (Strength): ti, ab, kw (Word variations have been searched) 46545 #9 Monolithic zirconia crowns 67 #10 3D printing 463 #11 Milling 191 #12 CAD CAM 513 #13 Zirconia 627 #14 Dental Ceramic 945 #15 Zirconia 627 #16 Dental Prosthesis 2182 #17 Digital one-piece casting 1 #18 3D printing using zirconia 1 #19 Biaxial flexural strength 13 #20 Colour infiltration 113 #21 Partial sintering 8 #22 Additive technology 196 #23 Dental crown 1366 #24 Direct inkjet printing 0 #25 Mechanical properties 2035 #26 Biomedical applications 306 #27 Marginal fit 338 #28 Digital impression 374 #29 Intraoral scanner 96	549

**Table 3:** Newcastle–Ottawa Scale showing quality assessment of studies.

Authors	Selection (up to 4 *)	Comparability (up to 2 *)	Outcome (up to 3 *)	Total	Interpretation
Batson et al. [20]	**		**	4/9	Moderate
Konstantinidis et al. [21]	***		***	6/9	Moderate
Tang et al. [22]	**		**	4/9	Moderate
Kitaoka et al. [23]	**		**	4/9	Moderate
Hansen et al. [24]	**	**	**	6/9	Moderate
Worni et al. [25]	**		***	5/9	Moderate
Gunge et al. [26]	**	**	**	6/9	Moderate
Zou et al. [27]	**		***	5/9	Moderate
Belli et al. [28]	**		**	4/9	Moderate

For the table above: 1 to 3 *—low quality; 4 to 6 *—moderate quality; 7 to 9 *—high quality of assessment.

**Table 4:** Summary of the data extracted from the studies included in the systematic review.

Article (Authors/Year of Publication)	Batson et al., 2014 [20]	Konstantinidis et al., 2018 [21]	Tang et al., 2019 [22]	Kitaoka et al., 2018 [23]	Hansen et al., 2018 [24]	Worni et al., 2017 [25]	Gunge et al., 2018 [26]	Zou et al., 2018 [27]	Belli et al., 2015 [28]
**Age**	Data not found	49.52	41.3	54	56.3	59.1	>20	37	Data not found
**Individuals (*n*)**	22	65	46	18	13	40	101	289	Data not found
**Zirconia Restorations (*n*)**	10	65	49	26	84	238	148	321	716
**Glaze/Stain (Yes or No)**	Yes	Yes	No	No	No	Yes	Yes	No	Data not found
**Location (Anterior/Posterior)**	Posterior	Posterior	Posterior	Posterior	Anterior	Posterior/Anterior	Posterior	Posterior	Posterior
**Presence/Absence of Plaque**	Data not found	Absence	Presence	Presence	Presence	Presence	Data not found	Data not found	Data not found
**Surface Treatment**	Data not found	Data not found	Final polishing	Data not found	Data not found	Data not found	Final polishing	Data not found	Data not found
**Marginal Integrity**	90%	93.80%	100%	88.46%	31.60%	100%	Data not found	98.80%	Data not found
**Bleeding on Probing (BOP)**	No alteration	1.80%	4.08%	No alteration	100%	10.60%	Data not found	Data not found	Data not found
**Color Stability (Yes or No)**	No alteration	No	Yes	No	Yes	No	Data not found	Yes	Data not found
**Dental Vitality (*n*)**	Data not found	19	Data not found	3	Data not found	0	0	0	Data not found
**Failures (*n*)**	Data not found	0	1	0	1	0	1	0	0
**Survival Rate**	Data not found	100%	93%	100%	98.81%	100%	91%	100%	100%

**Table 5:** In vitro studies that included the 3D printing of zirconium crowns.

Studies	Property Measured	Number of Ceramic Crowns	Techniques/Machines	Result	Conclusion
Wang, W. 2021 [6]	Dimensional accuracy and clinical adaptation	10	A conventional CAD–CAM system, X-MILL500 (XM) zirconia and 2 different stereolithography systems, CeraFab7500 (CF) alumina and CSL150 (CL) zirconia	CeraFab7500 (41 ± 11 mm) had better dimensional accuracy than CSL150 (65 ± 6 mm) or X-MILL500 (72 ± 13 mm) (P.05)	Better adaptation in the marginal, corner, and occlusal areas for X-MILL500 but reduced adaptation compared to CeraFab7500 and CSL150 (P.05) in the axial area
Wang, W. 2019 [7]	3D trueness	10	3D-printing system (CERAMAKER 900; 3DCeram Co) 5-axis, 2-bur milling machine (DWX-50; Roland DG Corp) for processing of the ZrO_2_ block (Zenostar; Wieland Dental)	The trueness values for both the systems had *p*-value less than 0.05	The trueness of all the surfaces of 3D-printed crowns equaled the trueness of the CAD-CAM crowns
Lerner, H. 2021 [5]	Marginal adaptation	10	(LCM) printer (Cerafab S65^®^, Lithoz, Vienna, Austria) 5-axis milling machine (DWX-52D^®^, DGShape, a Roland Company, Hamamatsu, Japan)	Median differences measured on margins and occlusal levels were 26.9 µm and 8.2 µm for printed and milled crowns, respectively	Statistically higher trueness in the milled crowns as compared to the 3D-printed ones
Kim, M.S. 2022 [8]	Microstructure, flexural strength, and fracture toughness	No Data	Milling machine (Zirkonzahn CAD/CAM System 5-TEC, Zirkonzahn), 3D printer (CeraMaker 900, 3DCeram)	The three-point flexural strength values of the Y-TZP ceramics produced by SM and SLA were 927 and 865 MPa, respectively	No significant changes in flexural strength (*p* = 0.242) or fracture toughness (*p* = 0.101)
Abualsaud, R. 2022 [9]	Internal fit, marginal adaptation, precision, and trueness	20	5-axis milling machine (PrograMill PM7, Ivoclar Vivadent, Schaan, Liechtenstein) 3D-printer (CERAMAKER C900 Flex, 3DCeram Sinto, Bonnac-laCôte, France	At the occlusal (8.77 0.89 m) and intaglio (23.90 1.60 m) surfaces of 3D-printed crowns, the highest and lowest trueness values were observed	Similarities existed between the internal and marginal fits of the two production methods
Abulsaud, R. 2022 [11]	Physiomechanical and surface properties	80	stereo-lithography using a 3D-printer (CERAMAKER C900 Flex, 3DCeram Sinto, France) Dry milling using a 5-axis milling machine (PM7)	The greatest and lowest reported densities were milled (6.056 0.116 g/cm^3^) and tilted (5.942 0.266 g/cm^3^), respectively	The biaxial flexural strength of the milled group (1507.27 ± 340.10 MPa) were significantly higher than those of the 3D-printed groups (*p* < 0.01)

## Data Availability

The data that support the findings of this study are available from the corresponding author upon reasonable request.
